# Framework of magnetostrain responsive Ni–Mn–Ga microparticles driving magnetic field induced out-of-plane actuation of laminate composite

**DOI:** 10.1038/s41598-023-33945-y

**Published:** 2023-05-03

**Authors:** DongKeun Han, Wan-Ting Chiu, Masaki Tahara, Volodymyr Chernenko, Senentxu Lanceros-Mendez, Hideki Hosoda

**Affiliations:** 1 Institute of Innovative Research (IIR), Tokyo Institute of Technology, Yokohama, 226-8503 Japan; 2 IRFI-WRH, Tokyo Institute of Technology, Yokohama, 226-8503 Japan; 3 University of Basque Country (UPV/EHU), 48080 Bilbao, Spain; 4 BCMaterials, Basque Center on Materials, Applications and Nanostructures, UPV/EHU Science Park, 48940 Leioa, Spain; 5 Ikerbasque, Basque Foundation for Science, 48009 Bilbao, Spain

**Keywords:** Materials for devices, Materials science, Ferromagnetism, Magnetic properties and materials

## Abstract

Ni–Mn–Ga single crystals (SC) exhibiting a giant magnetic field induced strain (MFIS), resulting from twin boundaries rearrangements, are excellent materials for novel actuators although enhanced brittleness and high costs are remaining the issues for applications. In polycrystalline state Ni–Mn–Ga alloys show small MFIS due to grain boundary constraints. By simple size reduction of the mentioned materials it is hardly possible to create quasi-two-dimensional MFIS actuators on the microscale with a pertinent out-of-plane performance. In pursuit of the trend for next generation materials and functions by design, in the present work we have developed a laminate composite as a prototype of microactuator with the out-of-plane stroke being driven by a framework of magnetostrain responsive Ni–Mn–Ga microparticles. The laminate consisted of the layer of crystallographically oriented Ni–Mn–Ga semi-free SC microparticles sandwiched between bonding polymer and Cu foils. Such design provided a particles isolation with a minimum constraint condition from the polymer. MFIS of the individual particles and the whole laminate composite was investigated by X-ray micro-CT 3D imaging. Both particles and laminate exhibited the same recoverable out-of-plane stroke produced by the particles´ MFIS of around 3% under 0.9 T. The developed microactuator design is promising for applications in the areas of micro-robotics, optical image stabilization in cameras, pumps for microfluidics etc.

## Introduction

Heusler type off-stoichiometric Ni–Mn–Ga alloys are well-known ferromagnetically ordered compounds undergoing a thermoelastic martensitic transformation (MT) from cubic austenitic phase to the modulated tetragonal, 5 M, or orthorhombic, 7 M, and to the non-modulated tetragonal, 2 M, martensitic structures, depending on the composition^1,2^. These materials, called ferromagnetic shape memory alloys (FSMAs), attract a considerable attention of researchers and engineers due to a giant magnetic field induced strain (MFIS) they reveal in a single crystalline form. MFIS of 6% and 12% under a magnetic field of the order of 0.1 T can be observed in the 5 M martensite, exhibited by the most to-date elaborated Ni–Mn–Ga compositions, and in the 2 M martensite of the heavily doped Ni–Mn–Ga analogs, respectively^3–8^. Such a huge magnetostrain effect (two orders of magnitude larger than a conventional magnetostriction) offers innovative technical solutions for the applications in actuators, sensors, vibrating energy harvesters etc.^8–13^ An underlying mechanism of the giant MFIS effect is a magnetic field-induced twin boundary motion^4,5,8^. This effect appears owing to both the strong magneto-elastic coupling and very low twinning stress in Ni–Mn–Ga FSMAs^14,15^. As a result, a magnetic field of the order of 0.1 T can exert an equivalent magnetostress (proportional to the magnetoelastic constant and square of the magnetic field) which is high enough (around 3 MPa) to move twin boundaries causing an increase in the fraction of magnetically favorable twin variants at the expense of less favorable ones^8^. This magnetically induced twin reorientation process is at the origin of aforementioned giant macroscopic strain. Depending on a twinning structure, Ni–Mn–Ga may exhibit an extremely low twinning stress, of the order of 0.1 MPa, so the reorientation of the martensitic twin variants even under a low magnetic field, entailing a reduced magnetostress, becomes possible^15,16^.

High fabrication costs and inherent brittleness are the main drawbacks of bulk Ni–Mn–Ga single crystalline actuators preventing their successful marketing. Addressing these problems, the efforts have been made in the literature to develop polycrystalline Ni–Mn–Ga analogs which actually failed to add any progress in FSMAs due to both the naturally higher brittleness than in the monocrystalline case and low MFIS values^17,18^. Low MFIS occurs because the mobility of the twin boundaries is limited by the geometrical constraints from the grain boundaries. The essential amount of grain boundaries can be eliminated in the Ni–Mn–Ga foams. Although foams showed a large initial MFIS, it was rapidly degraded during cycling^19^.

A recipe to remove grain boundaries in polycrystalline Ni–Mn–Ga alloys was radically advanced in our recent publications, where these alloys were carefully disintegrated, using specially elaborated intergranular fracture technology, into separate single crystalline grains exhibiting a large MFIS^19–25^. Such particles subsequently have been used to create bulk magnetostrain active Ni–Mn–Ga/polymer composites^21–23^. Here it is worth recalling that FSMAs show large work output, in the order of 10^7^ J m^−3^, and very favorable scaling behavior as far as this value tends to rise up to 10^8^ J m^−3^ with decreasing a sample size, all of those assure their multifunctional properties being retained on the microscale^26^. By contrast, small particles obtained from the bulk Ni–Mn–Ga alloys by the traditional methods, which involve highly damaging transgranular fracture, do not exhibit any practically important MFIS, whereby cannot be used for actuation on the microscale^27–29^.

Recent elaboration of the bulk composite materials consisting of the Ni–Mn–Ga single crystalline (SC) particles embedded in a silicone polymer, which have shown both a 4% of MFIS and a new phenomenon of the magnetic field induced rubber-like behavior, was a breakthrough in the about 20-years-efforts to create such materials^21,22,29–34^. There are several interrelated preconditions for a design of the magnetostrain active Ni-MnGa/polymer composites which were long time ignored. The most crucial conditions are two-fold: (1) individual microparticles should exhibit large MFIS, and (2) a stiffness-matched polymer should be used. Conditions (1) and (2) can be only realized if single crystalline microparticles plus polymer matrix with a stiffness of not more than 3 MPa are used. Fulfilling these conditions in Refs.^21,28^ enabled the aforementioned breakthrough in the FSMAs research area.

The unique magnetostrain properties, low-cost and easy production of the bulk Ni–Mn–Ga SC particles/silicone composites are characteristics which make these materials very attractive for novel actuator, sensing and haptic applications, where the value of stroke is more important than a generated force. If compared to the bulk single crystals, bulk composites contain no more than 30 vol% of particles embedded in a soft silicone polymer^21,24^, both factors obviously entail a reduction of the generated force. Another issue of the bulk composites is the problem of elastic interactions between the particles through a solid polymer whereby not all of them exhibit a magnetostrain^21,24^. By a simple size decrease of composite to the microscale all the aforementioned issues should become more pronounced which makes very doubtful their use as microactuators.

**Figure 1:**
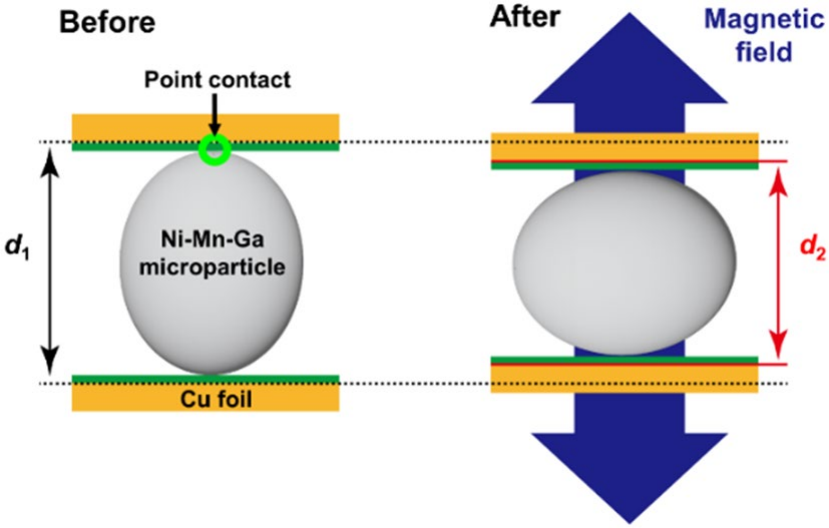
Schematic showing a concept of the magnetic field-induced actuation of Cu/Ni–Mn–Ga/Cu laminate composite driving by MFIS = (*d*_2 _− *d*_1_)/*d*_1_ effect exhibited by singe crystalline Ni–Mn–Ga microparticles.

To develop quasi-two-dimensional microactuators driven by the magnetic shape memory effect a new strategy is proposed in the present work. Contrary to the case of bulk particulate Ni–Mn–Ga SC/silicone composites, the Ni–Mn–Ga SC microparticles have been arranged in the controllable way as a functional layer which was sandwiched between Cu foils and fixed through small layers of silicone, serving as an adhesive, resulting at the end in a flat laminate composite. The concept of its functionality is schematically demonstrated in Fig. 1. Contrary to Ref.^23^, the actuator design shown in Fig. 1 can provide a maximum freedom for the shape changes of particles by excluding any constrains from the neighbors, and due to an absence of polymer matrix, in the ideal case, it allows to increase the areal density of the non-touching spheres up to 85% taking into account their maximum possible shape change of 6%. The magnetic field-induced cooperative shape changes of the particles in the functional layer generate the out-of-plane stroke of the laminate as illustrated in Fig. 1. In this case, it is implied that a major part of the particles have been already crystallographically oriented during curing under the in-plane magnetic field providing that the easy-magnetization short *c*-axes of tetragonal unit cells are aligned in-plane of a functional layer with the long *a*-axes perpendicularly to it. After application of the magnetic field normally to the surface of composite, the unit cells in the particles become reoriented with their short axes along magnetic field direction (as a result of the ferromagnetic shape memory effect), whereby the actuator exhibits contraction as shown in Fig. 1. To restore the original thickness of actuator, the magnetic field should be applied in the transversal direction, i.e., along the composite plane. Thus, the periodic stroking of actuator can be achieved in the rotating magnetic field or by alternating switching of the orthogonally mounted magnetic field sources^13,35^. These microactuators can be implemented in the devices for micro-robotics, optical image stabilization in cameras, pumps in microfluidics etc.

## Results

### Structure and transformation behavior of single crystalline Ni–Mn–Ga particles

**Figure 2:**
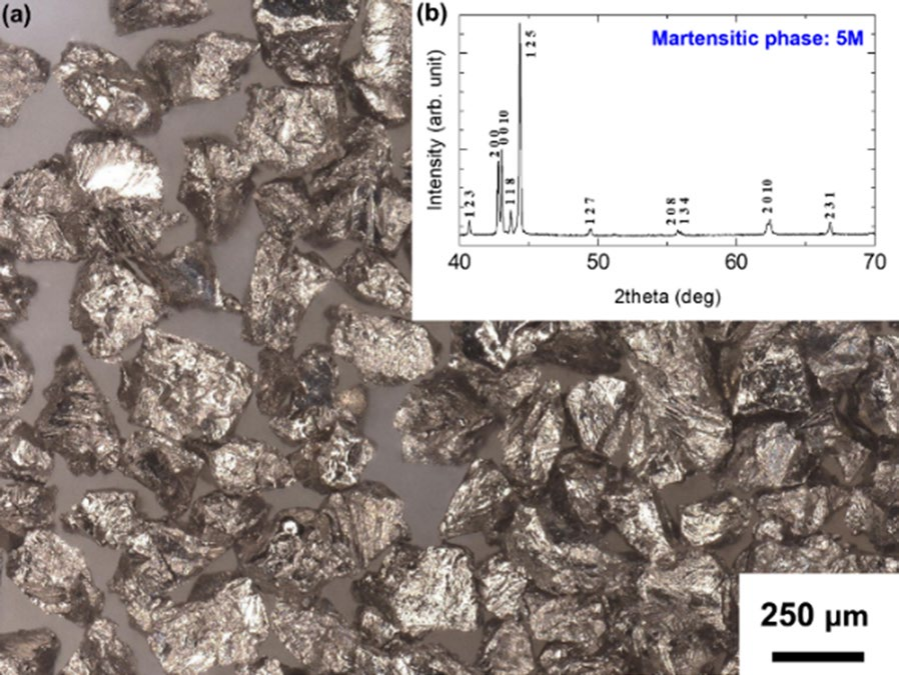
(**a**) Optical image of single crystalline Ni–Mn–Ga microparticles. (**b**) X-ray diffraction pattern of the Ni–Mn–Ga particles measured at room temperature.

The magnetostrain active particles representing mostly single crystalline grains faceted by the atomic planes with a minimum of the crystal defects were prepared using Ni_50_Mn_28_Ga_22_ + Bi_0.03_ (at%) polycrystalline alloy as described in section Methods. Bi atoms fully segregate at the grain boundaries, thereby radically increasing an intergranular brittleness. After careful mechanical cleaving of the polycrystalline ingot and subsequent heat treatment, the particles were divided into several size fractions. The major 150–250 μm size fraction, shown in Fig. 2a, was selected for DSC characterization and for a manufacture of the microactuator prototype. The fraction below 45 μm was used for a precise powder X-ray diffraction (XRD) measurements at the room temperature. Figure 2b depicts XRD pattern where all reflections are indexed in terms of 5 M-modulated slightly monoclinic martensitic phase with the lattice parameters of *a*_m_ = 4.21 Å, *b*_m_ = 5.60 Å, and *c*_m_ = 20.90 Å. Disregarding modulation, the martensitic lattice in the cubic coordinate system is well approximated by the tetragonal unit cell with the lattice parameters *a*_M_ = 5.94 Å and *c*_M_ = 5.60 Å which is in good agreement with the literature^2,36^. The theoretically maximal MFIS value of the single crystalline particle can be evaluated using expression:

1 $$\varepsilon _{{{\text{MFIS}}}} = \left( {1 - \frac{{c_{{\text{M}}} }}{{a_{{\text{M}}} }}} \right) \times 100\%$$

According to Eq. (1) and indicated parameters, the magnetic field (or mechanical stress)-induced conversion of one martensitic variant into another in the studied alloy should result in the uniaxial strain of 5.6%.

**Figure 3:**
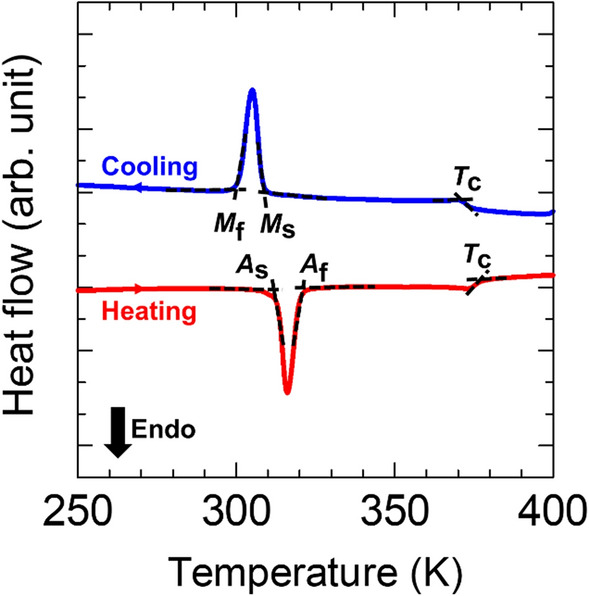
DSC heating–cooling curves of the single crystalline Ni–Mn–Ga particles. The characteristic temperatures of the forward start and finish MT (*M*_s_, *M*_f_) and reverse start and finish MT (*A*_s_, *A*_f_) and Curie temperature (*T*_C_) are indicated.

Calorimetric DSC heating/cooling curves, depicted in Fig. 3, show a transformation behavior of the particles through the exothermic and endothermic peaks corresponding to the forward and reverse MT, respectively. The characteristic temperatures of MT have been determined by a standard two-tangent method to be *M*_s_ = 309 K and *M*_f_ = 300 K for the start and finish temperatures of the forward MT, and *A*_s_ = 312 K and *A*_f_ = 320 K for the start and finish temperatures of the reverse MT. A kink at 373 K corresponds to the Curie temperature.

### X-ray micro-CT 3D imaging and magnetic properties of laminate composite

**Figure 4:**
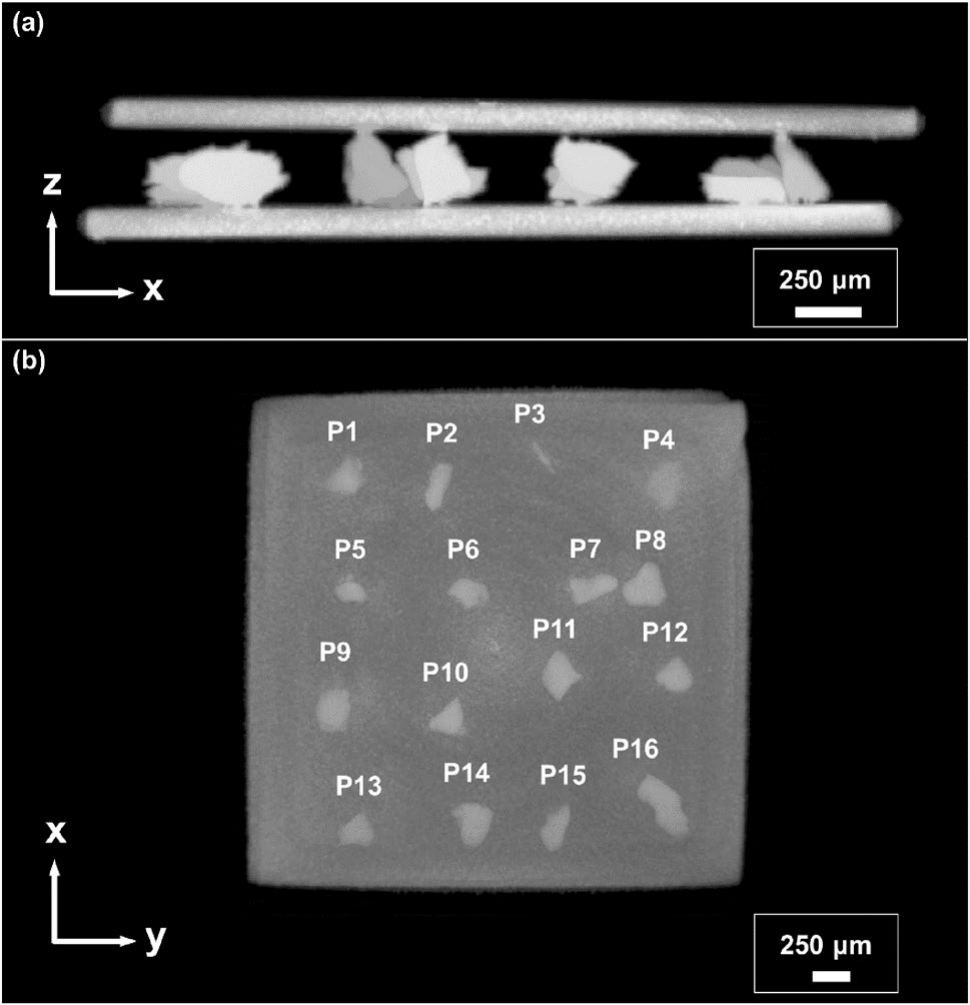
X-ray micro-CT 3D images of the Cu/Ni–Mn–Ga particles/Cu laminate composite: (**a**) side view and (**b**) top view.

Taking into account a design concept shown in Fig. 1, a prototype of the magnetostrain active laminated actuator consisting of the 16 Ni–Mn–Ga single crystalline particles, sandwiched between silicone polymer layers and Cu foils, was fabricated as described in Methods. The amount of particles was optimal in our experiment to ensure an actuation ability of the laminated composite and to optimize the time for a micro-CT analysis of all particles. X-ray micro-CT 3D images of the side and top views of the laminated composite are shown in Fig. 4. The silicone polymer was used as an adhesive which was chosen due to its sufficient time for curing at room temperature, whereby allowing the orientation of particles with the *c*-axes along the magnetic field applied during curing in the *x*-direction (Fig. 4). The Cu foil was selected because of its diamagnetic nature and high stiffness. Laminated composite has dimensions of 3 × 3 × 0.5 mm^3^. Its size can be easily reduced by 3 times, at least. The images in Fig. 4 confirm an intended arrangement of the Cu foil and particles. Depending on size and shape, particles appeared to be either attached to the Cu foils on both sides through the polymer layers of about 25 $\upmu$m thick (invisible in Fig. 4) or attached only on one side. The particles are numerated as shown in Fig. 4b. For the purposes of classification, particles attached to both sides are defined as 'effective particles' and those attached to one side are defined as 'non-effective particles'. Thorough observations show that the driving layer has six effective particles which contribute to the magnetically induced stroke of laminate.

**Figure 5:**
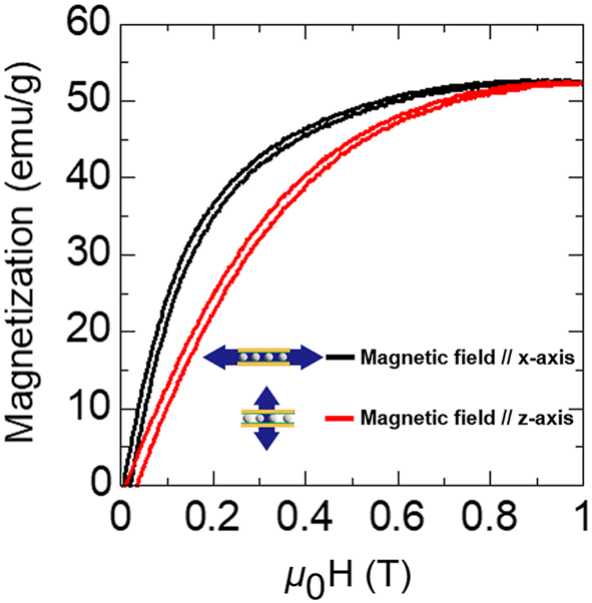
Magnetization curves of Cu/Ni–Mn–Ga particles/Cu laminate composite. Double arrow shows the direction of applied magnetic field with regard to the coordinate axes shown in Fig. 4.

Figure 5 displays the magnetization curves for the laminate measured with a field applied along *x*-axis, which was an in-plane curing direction, and along *z*-axis (normally-to-plane). When a magnetic field is applied in-plane of the laminated composite, particles reached the magnetic saturation much faster compared to the case when the magnetic field was applied normally-to-plane. The in-plane soft magnetic behavior confirms a preferable alignment of the easy-magnetization *c*-axes of particles along the magnetic field during curing, by virtue of that the hard-magnetization *a*-axes are aligned normally-to-plane of composite. The difference between easy and hard magnetization curves reflects the total magnetic anisotropy of the ensemble of particles in the composite. Magnetocrystalline anisotropy in Ni–Mn–Ga alloys is a prerequisite for the observation of large MFIS (see, e.g., Refs.^14,37^). Furthermore, Fig. 4 shows that saturation magnetization of the martensitic particles is equal to ~ 50 emu/g in the field close to 0.8 T, in correspondence with the typical values for bulk Ni–Mn–Ga alloys at 298 K^38–40^.

### Magnetostrain behavior of laminated composite and individual particles

**Figure 6:**
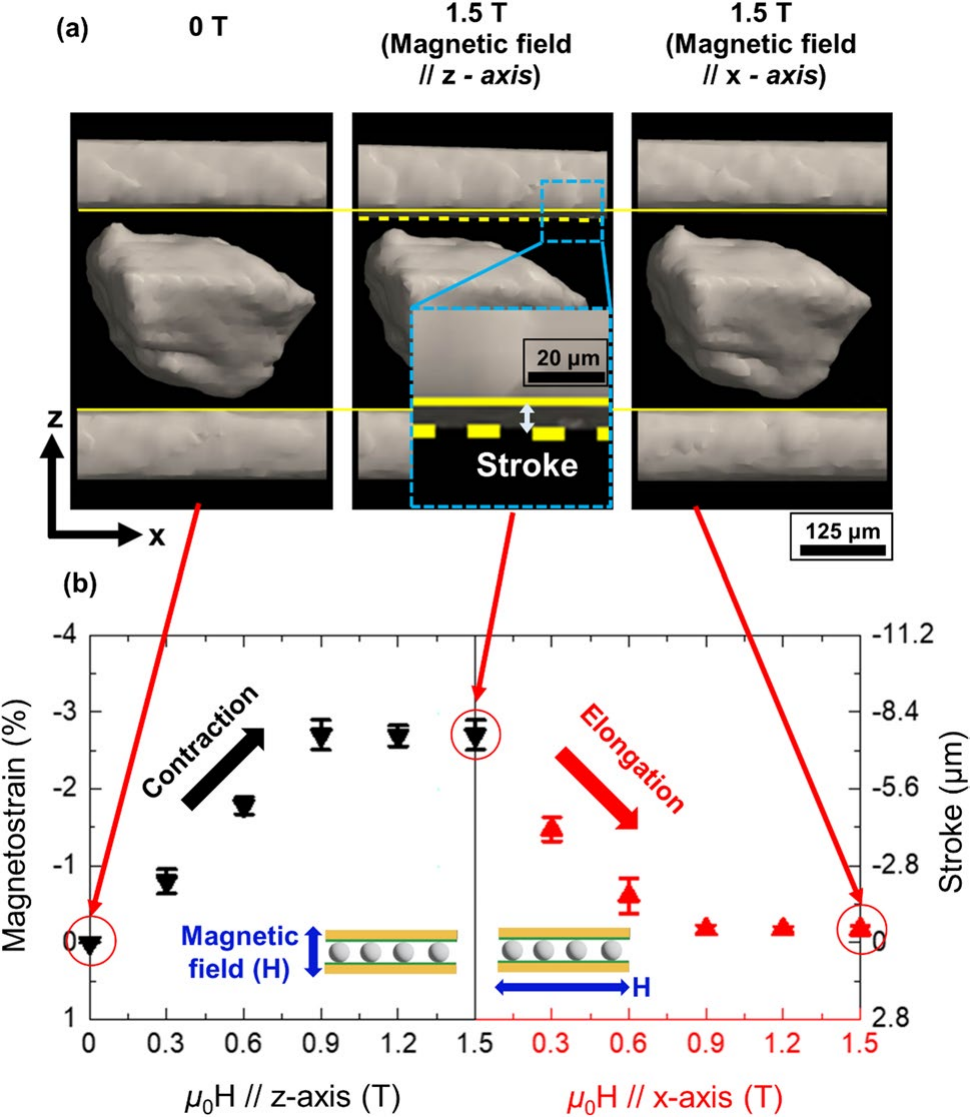
Magnetic field induced thickness change of Cu/Ni–Mn–Ga particles/Cu laminate composite: (**a**) X-ray micro-CT 3D images of the in-plane sectional view showing a decrease and increase of the composite thickness produced by the magnetostrain response of the effective particles under in-plane and normally-to-plane magnetic field. The maximum stroke is zoomed for better visibility. (**b**) Magnetic field dependences of the out-of-plane strain of the composite.

Magnetic field induced out-of-plane strain of the laminated composite (stroke along *z*-axis), as well as MFIS of all individual particles along *z*-axis have been studied by the analysis of the X-ray micro-CT 3D images obtained under magnetic field. Figure 6a presents a section view of Cu/Ni–Mn–Ga particles/Cu laminated composite showing a decrease and increase of the composite thickness produced by the magnetostrain response of the effective particles under differently oriented magnetic field. As already described, the initial composite thickness was set during its curing (see image at 0 T corresponding to a zero magnetostrain in Fig. 6b). The step-like application of the magnetic field along *z*-axis entails contractions of the particles until the saturation of both MFIS and magnetization at 0.9 T are achieved (see also Fig. 5). Magnetic field increments give rise to a decreasing dependence of the composite thickness until the mentioned saturation conditions for the particles are achieved resulting in a maximum negative stroke of about 8 μm and corresponding magnetostrain equal to 2.7% (see the image in Fig. 6a with the inset illustrating a zoomed area and the contraction curve in Fig. 6b). After removal of a magnetic field the accumulated strain is retained because there is no force which can drive the composite thickness to its initial state. A recovery stress, indeed, appears when the particles begin to elongate along *z*-axis under the magnetic field applied along the *x-*axis (see the elongation curve in Fig. 6b). As a result, a full restoring of the composite thickness occurs at the same field value of 0.9 T and further field increment does not have an effect (see third image in Fig. 6a and the elongation curve in Fig. 6b).

To assess the magnetostrain of each of the individual 16 particles, we conducted a quantitative evaluation of their field induced deformation along *z*-axis using a special procedure, see section Methods. Under increasing/decreasing the magnetic field all particles show similar qualitative dependences as the ones depicted in Fig. 6b. Actually, the effective particle 8 shown in Fig. 6a, as well as effective particles 7, 12, and 16 shown in Supplementary Fig. S1, exhibit exactly the same parameters of MFIS as the entire composite. Supplementary Figs. S3–S6 summarize the results of magnetic field-induced microdeformation for all the particles, justifying that all of them individually exhibit a high MFIS.

The effective particles drive the magnetic field induced actuation of composite reaching up to 8 μm of stroke with the corresponding magnetostrain value equal to about 2.7%. A rather good reproducibility of the composite stroke measured at 1 T was checked 10 times in the present work. The results of the cycling measurements are presented in Supplementary Fig. S7.

Likewise effective microparticles, the non-effective ones, e.g., microparticles 1, 4, 5, 13 in Supplementary Fig. S2, also exhibit a large micro-magnetostrain, but inasmuch as they are not in a direct contact with the upper Cu plate through a polymer, they do not contribute to the laminate performance.

## Discussion

Motion technologies based on the magnetic actuation may play an important role in developing novel MEMS since a magnetic field has unique advantage in controlling motion inside of an enclosed space. The magnetostrain results depicted in Fig. 6 and Supplementary Figs. S1–S6 confirm this assertion. Inside of the laminate composite, the Ni–Mn–Ga microparticles exhibit a magneto-mechanics typical for the bulk single crystal. Moreover, the magnetostrain behavior of the entire composite copies the one for both the individual particle (Fig. 6) and for the particles ensemble. The full analogy goes not only for the similar MFIS values, but also for other characteristics such as, e.g., dissymmetry in the contraction/elongation rates observed in the rotation experiments^35^ at the moments when the magnetic field direction changes by 90 degree. For instance, it is easy to see that the left and right dependences in Fig. 6b show different values of magnetostrain at 0.6 T. This dissymmetry can be explained in the following way. According to the laminate design, it consists of the 250-micron-thick layer of particles which is fixed between Cu plates by two 25-micron-thick layers of silicone polymer so, only about fifth part of volume fraction of effective particles are stuck into the polymer layers along *z*-axis. Accordingly, along this direction the polymer layers with elastic stiffness of around 0.5 MPa^21^ could resist against the extension/contraction of particles, whereas in *x*-direction the particles have a freedom to deform. It has to be recalled that the twinning structure of particles was set during composite curing under a magnetic field applied along *x*-direction. After composite curing, the twinning structure should be in a stress-free state, where a volume fraction of twin variants with *c*-axis along *x* direction is dominated. It is reasonable to assume that in the process of application of the magnetic field along *z* direction the twin rearrangement would be slower, due to elastic resistance of the polymer, than during its return to the initial stress-free twinning structure under magnetic field applied in *x*-direction. In other words, it was easier to deform composite when the magnetic field applied in *x* direction than along *z* direction, which could be a reason for the dissymmetry in the MFIS evolutions observed in Fig. 6.

As already indicated, the lattice parameters of tetragonal martensitic structure (precisely, their *c/a* ratio) of the studied particles in the cubic coordinate system allow obtaining a maximum theoretical strain of 5.6% in the case when only one twin variant is reoriented into another. Whereas the formation of only one twin variant in the sample is practically possible in Ni–Mn–Ga single crystals of the regular shape after their special training treatments (see Refs.^6–8^ and references therein), this is not obvious for the irregularly shaped particles which are likely contain more than two twin variants even after composite curing under a magnetic field. Moreover, one should not discard the possibility of constraint influence of the polymer layers on some volume fraction of the twin variants in each particle. A magnetic field application can only move twin boundaries between the favorably oriented twin variants. Since the latter ones comprise a part of the sample volume, the particles exhibit MFIS values below the theoretical limit, as observed in our experiments. Nevertheless, measured MFIS values, of about 3%, are still very large if compared with the ordinary magnetostriction^8^. What is also important for engineering applications that MFIS is fully recoverable under consecutive change of the magnetic field direction, see contraction and elongation dependences under applied differently oriented magnetic field in Fig. 6 and Supplementary Figs. S1 and S2. This means that the developed composite can be effectively activated in the rotation magnetic field or by alternating switching of the orthogonally mounted magnetic field sources. Furthermore, one should emphasize that alongside the large recoverable magnetostrain our composite generates a reverse stress equal to 1 × 10^−2^ kPa by lifting a 0.011 g which is the weight of polymer layer plus Cu foil. Special research, including finite elements modeling, is needed to examine the influence of the type and thickness of polymer layers and amount of effective particles on the reverse stress of the developed actuator prototype.

In summary, a prototype of magnetostrain active laminated actuator consisting of the Ni–Mn–Ga single crystalline microparticles framework, sandwiched between silicone polymer layers and Cu foils, was fabricated in the present work. Such an actuator design was a key factor which excluded constraints among the particles and minimized constraints from the polymer layers whereby facilitating large MFIS values of all individual particles. The magnetization and magnetostrain behavior of the newly designed Cu/Ni–Mn–Ga particles/Cu laminate composite were investigated using magnetization curves and X-ray micro-CT 3D imaging analysis, respectively. It was experimentally confirmed that the cooperative MFIS´s of microparticles drive field-induced actuation of laminate composite. As a result, the quasi-two-dimensional composite exhibited the out-of-plane actuation under a magnetic field of 0.9 T with a recoverable stroke of 8 μm (of about 3% of the particles magnetostrain) generating a reversion stress of 1 × 10^−2^ kPa. A possible maximum force output which can be generated by the 6 active particles in the laminated composite can be roughly estimated if 1 MPa is accepted as the magnetostress limit which can be produced by a single crystalline Ni–Mn–Ga^8^. Taking into account the average value of the cross-section of $\sim 0.25$ mm for a particle, one can obtain a maximum output force equal to $\sim 0.5$ N for 6 particles.

These findings suggest that the laminate design facilitates the application of Ni–Mn–Ga particles in micro-sized actuators suitable for novel magnetically activated devices in micro-robotics, optical image stabilization in cameras, pumps in microfluidics etc.

## Methods

### Fabrication of microparticles and their basic characterization

The technological details of the preparation of single crystalline particles of Ni–Mn–Ga FSMAs can be found in Refs.^21–23^. A main know-how in this case consists in adding a tiny amount of Bi which fully precipitates on the grain boundaries of the alloy making it very brittle. A polycrystalline ingot with the chemical composition of Ni_50_Mn_28_Ga_22_ + Bi_0.03_ (at%) was fabricated by arc melting under an Ar-1 vol%H_2_ atmosphere using high-purity Ni (99.99%), Mn (99.9%), Ga (99.9999%), and Bi (99.999%). The ingot was flipped and re-melted six times. It was then homogenized at 1273 K for 24 h followed by ice water quenching. The mechanical disintegration of the ingot into separate single crystalline grains occurred in a state in which the binding force of the grain boundaries in the alloy were significantly weakened, that was at enhanced temperatures where Bi becomes liquid. As a result, it was possible to obtain the irregular shape single crystal-like particles with clean surfaces. To remove residual stress and improve atomic order, the grain particles were sealed in an evacuated quartz tube, heat-treated at 1073 K for 1 h, and furnace cooled. After heat treatment, the particles were divided into size fractions using a sieve set. Particles observation was undertaken using an optical microscope (OM-VHX7000).

The structure identification was carried out by X-ray diffraction (XRD-PANalytical X’pert Pro Galaxy, Philips) with Cu–Kα (λ = 1.5405 Å) radiation in a 2*θ* range from 20° to 120° at 298 K. Si powder was used as a standard to correct the systematic error in the XRD measurements. To ensure high reliability of the X-ray experiment, the Ni–Mn–Ga particles fraction of less than 45 μm were used.

The MT transformation temperatures (*M*_s_, *M*_f_, *A*_s_, *A*_f_) and Curie temperature (*T*_c_) were determined by the standard two-tangent method using differential scanning calorimetry curves measured with a DSC-60 Shimadzu instrument. DSC dependencies were recorded during heating and cooling ramps (10 K/min) in the temperature range of 243–423 K.

### Fabrication and characterization of laminate composite

**Figure 7:**
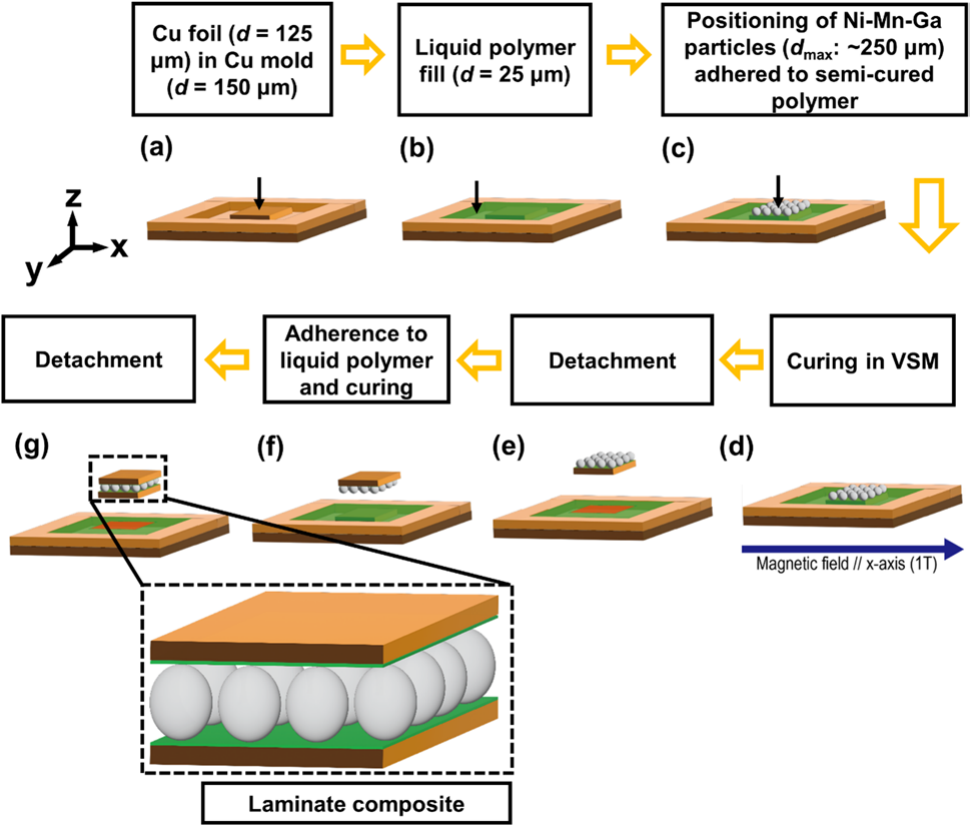
Schematic of a fabrication process flow of Cu/Ni–Mn–Ga/Cu laminate composite comprising a layer of single crystalline Ni–Mn–Ga microparticles adhered to the Cu foils (orange color) by a silicone polymer layer (green color).

Figure 7 presents a schematic of the fabrication process flow of Cu/Ni–Mn–Ga/Cu laminate composite. A pure copper (99.99%) foil with a thickness *d* = 125 μm was cut into a size of 3 mm × 3 mm using an electric discharge machine. A polished Cu mold with a square-shaped recess of 150 μm deep was then set as a guide (Fig. 7a). The commercial silicone rubber, ELASTOSIL M4400 and Waker A.G. polymer and curing agent T40 Waker Catalyst were mixed at a ratio of 100:3 and uniformly filled the copper mold up to its brim level (Fig. 7b). The polymer was then cured at room temperature for 4 h until it was in a semi-liquid condition (Fig. 7c). This semi-liquid polymer layer of 25 μm thick was served as an adhesive. The 16 particles were then arranged in a polymer bed in a 4 × 4 manner and cured for 4 h in a magnetic field of 1 T applied an in-plane direction (Fig. 7d) to facilitate the orientation of the twin variants in the particles with their easy-magnetization short *c*-axes along the magnetic field direction. The same bonding procedure was performed with an opposite side of the laminate (Fig. 7e,f) before finally being detached from the mold (Fig. 7g).

Magnetization curves of composite were measured at room temperature using a vibrating sample magnetometer (VSM, TM-VSM1530-HGC-D, Tamakawa Co., Ltd.). Pure Ni sample was used as a standard to correct the systematic error in the VSM measurements.

Magnetostrain values of the composite and individual particles were obtained by X-ray micro-CT 3D images and their analysis with a software Molcer Plus (White-rabbit Co. Ltd) using visualization and processing through polar decomposition, respectively. The images were acquired with X-ray micro-computed tomography (Comscan Scan Xmate X-ray μCT) under 100 kV, 100 μA with 2 × 2 binning mode and 1200 projections, and the target distance was 3 × 21. The 3D images were obtained at a resolution of approximately 3 μm/pixel. For each imaging, the particles and respective composite were deformed ex situ under a magnetic field up to 1.5 T applied normally-to-plane or in-plane of flat composite by VSM with the steps of a 0.3 T. To confirm whether the laminated composite maintained the magnetostrain after removing the magnetic field, an *in-situ* X-ray micro-CT experiment was conducted under magnetic fields between 0.3 and 0.6 T produced by the permanent magnets.

The composite stroke resulting from the micromagnetostrain of particles layer was calculated as (see also Fig. 1):

2 $$\text{stroke }= {\text{d}}_{1}-{\text{d}}_{\text{i}}$$

where *d*_1_ and *d*_i_ represent the distance between Cu plates in the initial state and after application of the magnetic field (*i* = 0.3 T, 0.6 T, 0.9 T, 1.2 T, and 1.5 T), respectively. The mean value and standard deviation of the stroke (and calculated strain) were obtained for each deformed state of composite as a result of 200 measurements of the distance between 3D-imaged Cu plates. The X-ray micro-CT images were also used for a quantitative evaluation of the microstrain of individual particles. The deformation gradient was calculated from the coordinates obtained from a specific point of the particles, and analyzed through polar decomposition, for details see Refs.^21–23^. For each particle and its deformed state, three rounds of the processing through polar decomposition were used to evaluate the error bars in the micromagnetostrain curves.

## Supplementary Information

Supplementary Figures.

## Data Availability

Authors confirm that all relevant data were included in the article and/or in the supplementary information files.
