# Supplementary material for: Framework of magnetostrain responsive Ni–Mn–Ga microparticles driving magnetic field induced out-of-plane actuation of laminate composite
Source: Sci Rep. 2023 May 3;13:7160. doi: 10.1038/s41598-023-33945-y (PMC10156658; doi:10.1038/s41598-023-33945-y)
Supplement: Supplementary file 1 — Supplementary Figures. [file 41598_2023_33945_MOESM1_ESM.pdf]

Supplementary materials

Framework of magnetostrain responsive Ni-Mn-Ga microparticles driving magnetic field induced out-of-plane actuation of composite laminates

Dong Keun Han, Wan-Ting Chiu, Masaki Tahara, Volodymyr Chernenko*, Senentxu

Lanceros-Mendez and Hideki Hosoda*

Figures S1 – S6. Micro-magnetostrain and deformation exhibited by each of the 16 individual particles along *z*-axis under magnetic field applied out-of-plane (along *z*-axis) and in-plane (along *x*-axis) of laminated composite.

Figure S7. Cyclic behavior of the magnetic field induced out-of-plane stroke of the laminated composite.

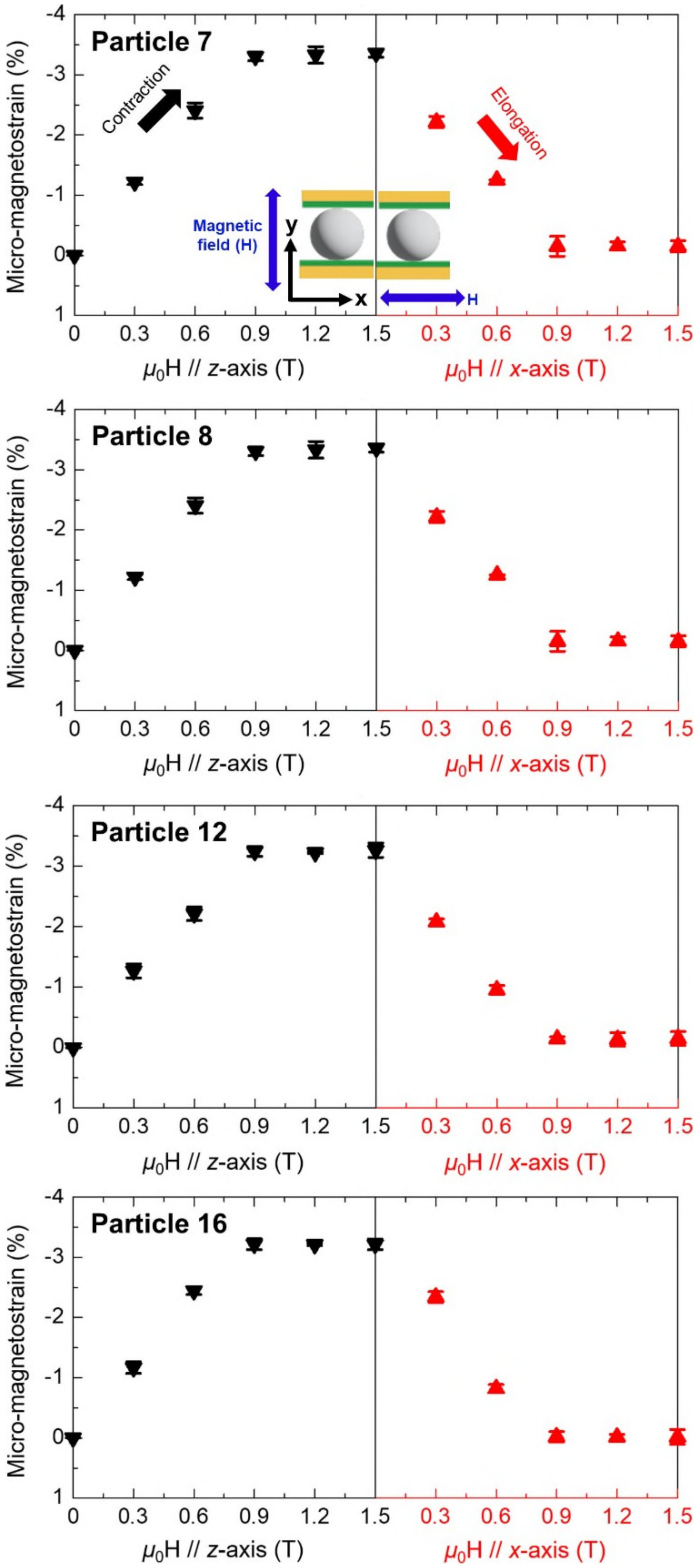

Figure S1. Magnetostrain along z-axis of the ´effective´ microparticles 7, 8, 12, and 16 as a function of magnetic field.

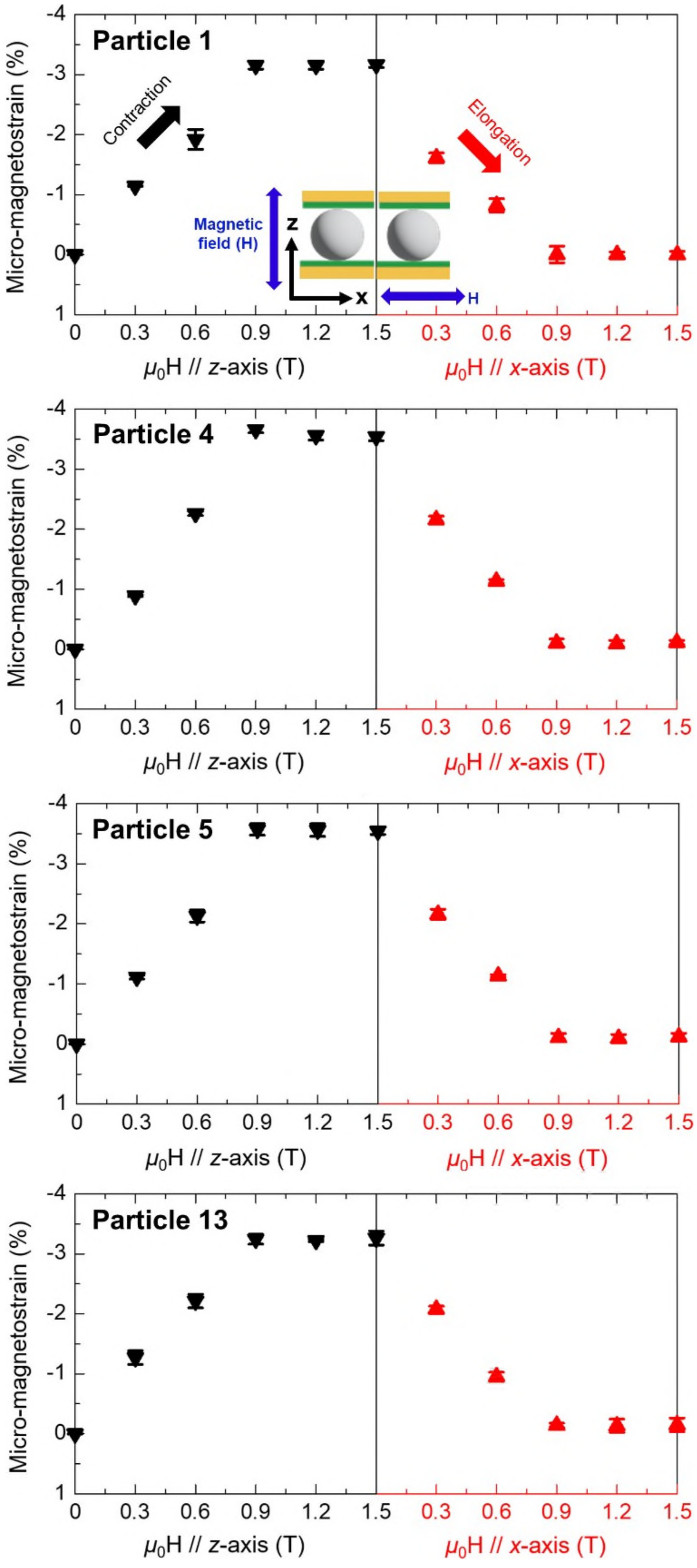

Figure S2. Magnetostrain along z-axis of the ´non-effective´ microparticles 1, 4, 5, and 13 as a function of magnetic field.

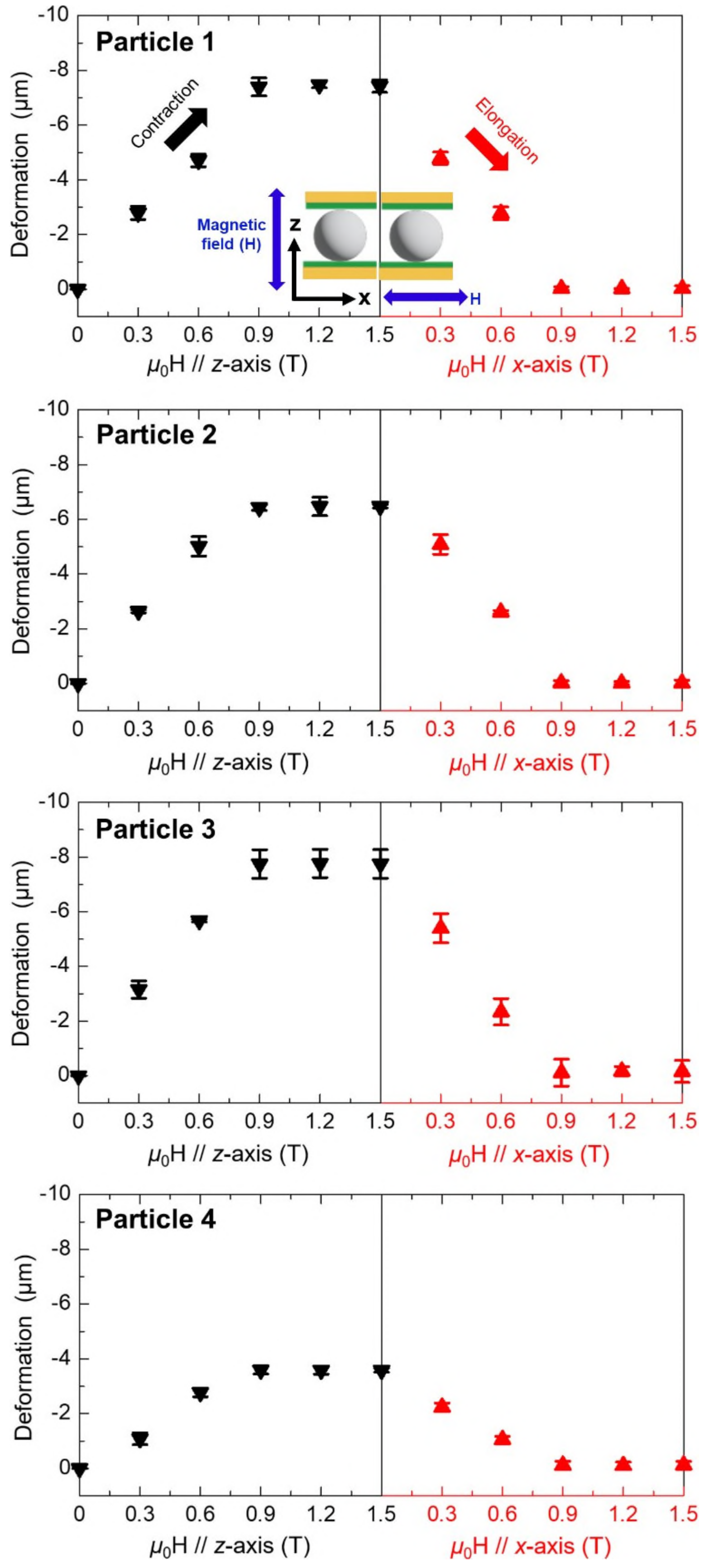

Figure S3. Magnetic field induced deformation along z-axis of particles 1, 2, 3, and 4.

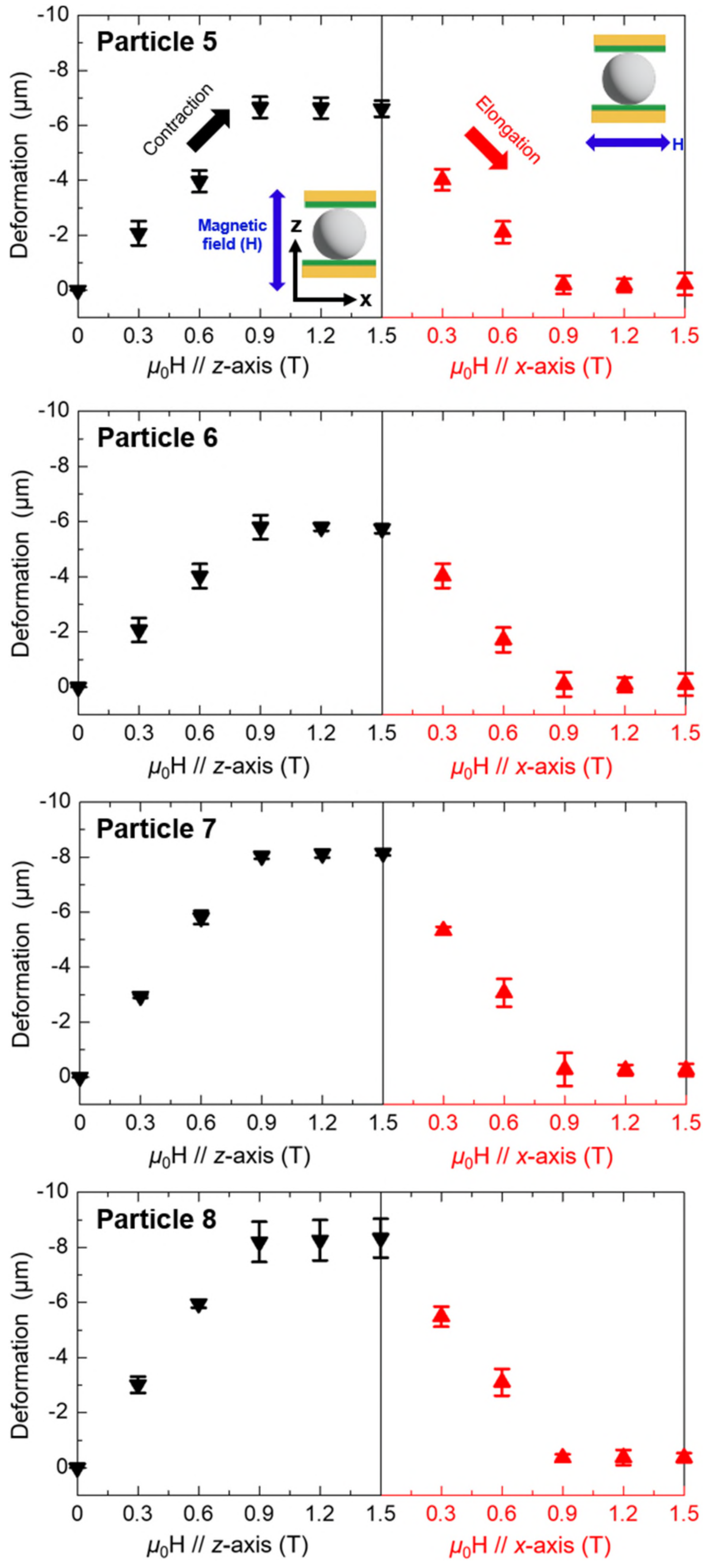

Figure S4. Magnetic field induced deformation along z-axis of particles 5, 6, 7, and 8.

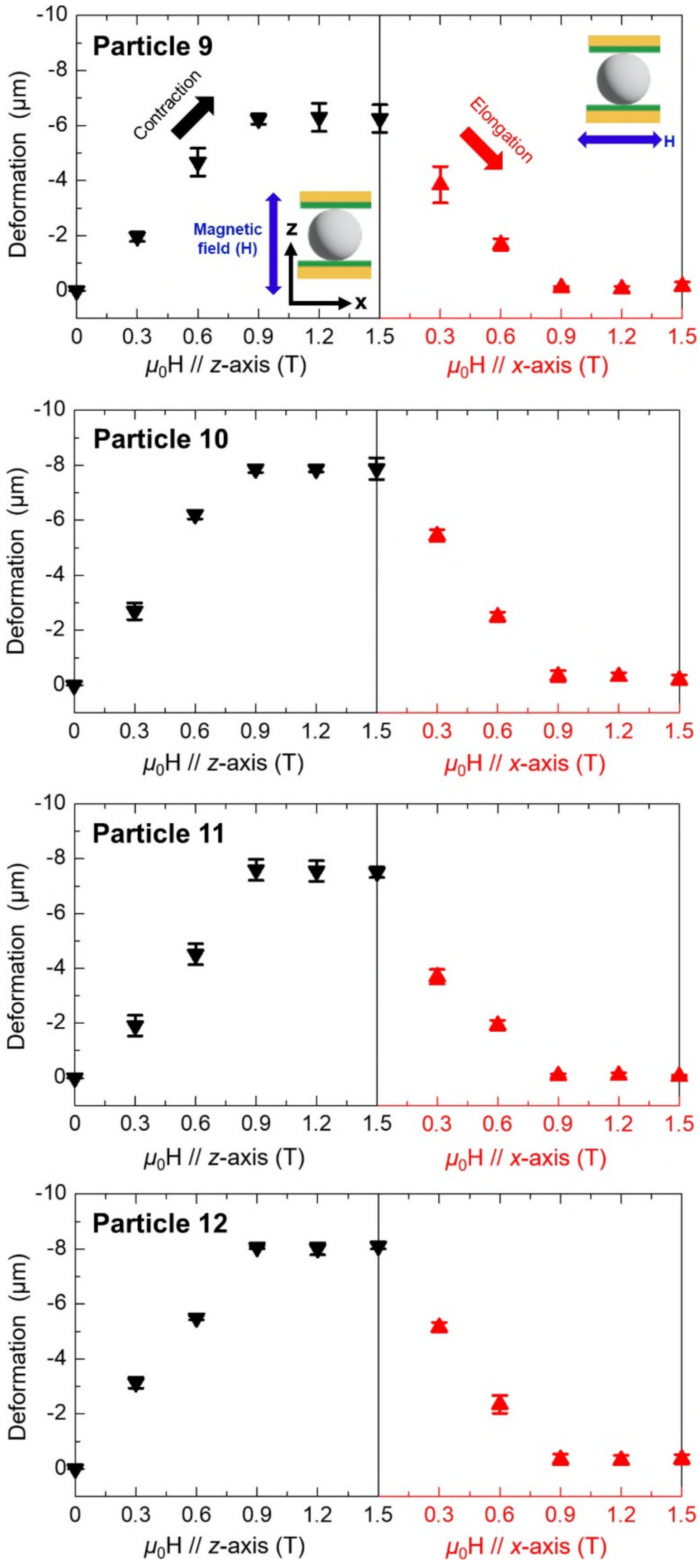

Figure S5. Magnetic field induced deformation along z-axis of particles 9, 10, 11, and 12.

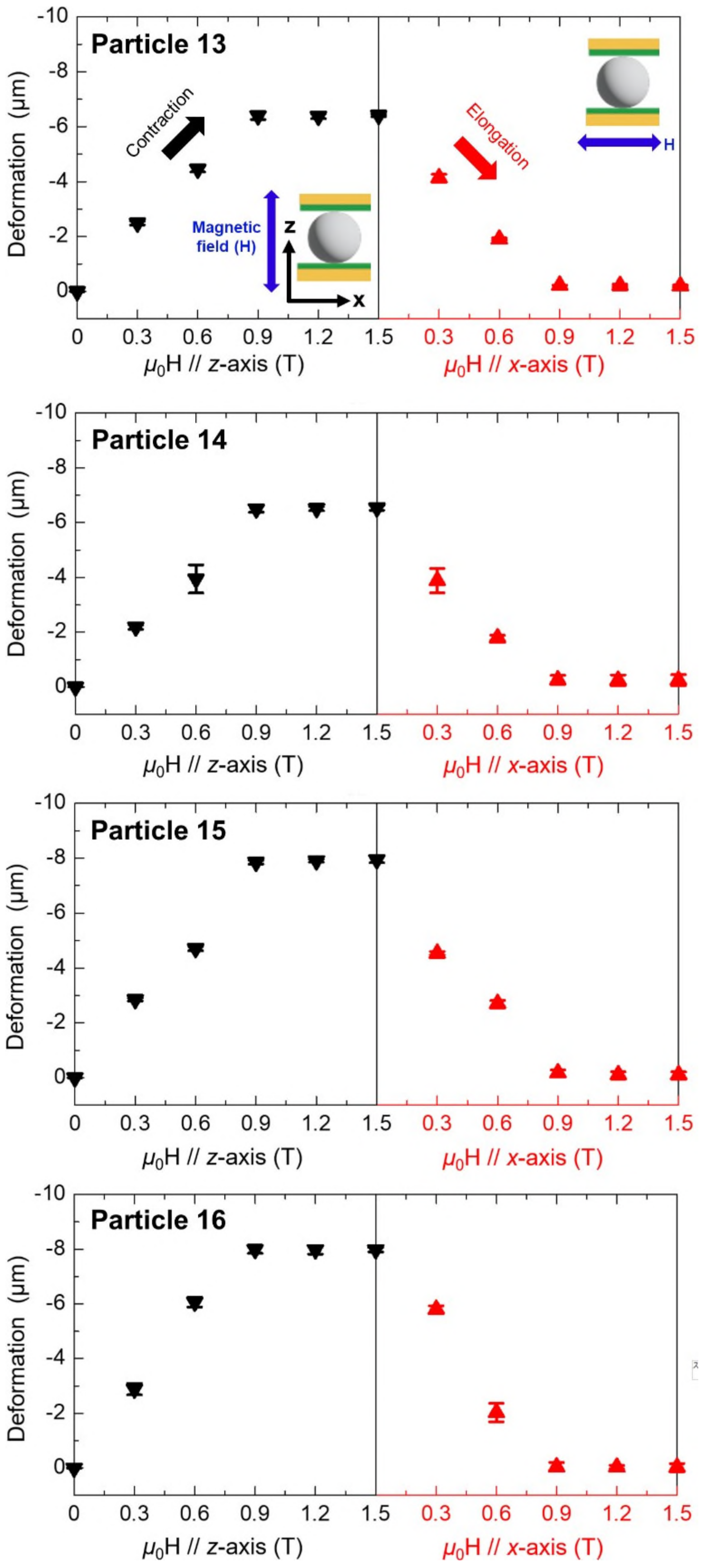

Figure S6. Magnetic field induced deformation along z-axis of particles 13, 14, 15, and 16.

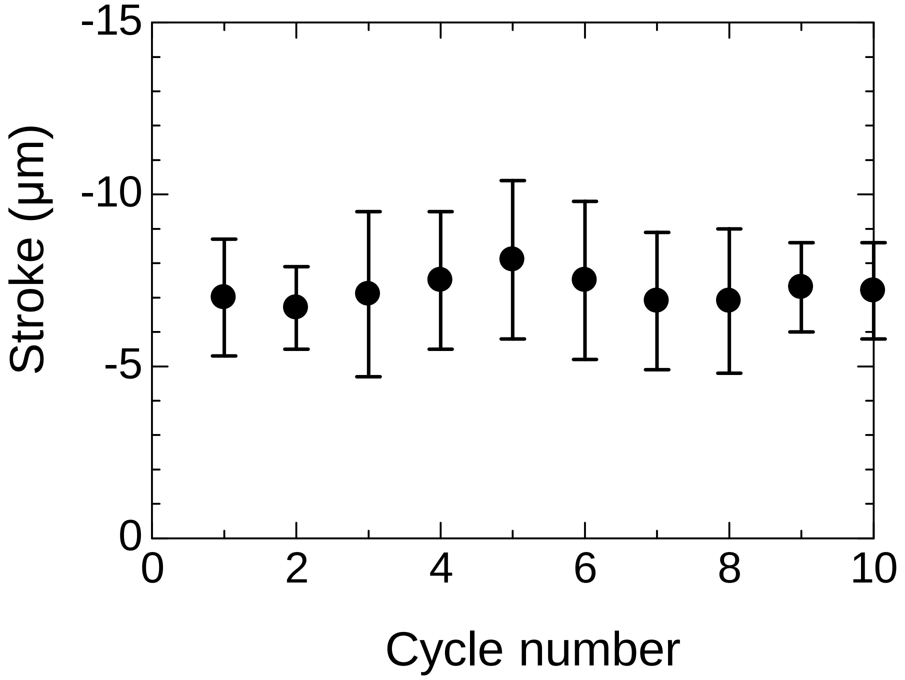

Figure S7. Cycling of the magnetic field induced out-of-plane stroke of the laminate composite. The data shown in the figure are contraction values of composite obtained under magnetic field of 1 T in *z*-direction. Before each measurement, the thickness of composite was reset by the magnetic field of 1 T applied along *x*-direction of composite.

Stroke measurements were performed at a resolution of 1µm by the thickness evaluation focus-defocus method using 3D imaging of a digital optical microscope VHX7000 (Keyence Co.) .
